# Screening for frequent hospitalization risk among community-dwelling older adult between 2016 and 2023: machine learning-driven item selection, scoring system development, and prospective validation

**DOI:** 10.3389/fpubh.2024.1413529

**Published:** 2024-11-27

**Authors:** Eman Leung, Jingjing Guan, Qingpeng Zhang, Chun Cheung Ching, Hiliary Yee, Yilin Liu, Hang Sau Ng, Richard Xu, Hector Wing Hong Tsang, Albert Lee, Frank Youhua Chen

**Affiliations:** ^1^Department of Management Sciences, City University of Hong Kong, Kowloon, Hong Kong SAR, China; ^2^JC School of Public Health and Primary Care, The Chinese University of Hong Kong, Shatin, China; ^3^Epitelligence, Hong Kong, Hong Kong SAR, China; ^4^Department of Pharmacology and Pharmacy, HKU Musketeers Foundation Institute of Data Science, The University of Hong Kong, Pokfulam, China; ^5^Department of Rehabilitation Sciences, The Hong Kong Polytechnic University, Hung Hom, Kowloon, Hong Kong SAR, China; ^7^People Service Centre, Kowloon, Hong Kong SAR, China; ^6^Mental Health Research Centre, The Hong Kong Polytechnic University, Hung Hom, Kowloon, Hong Kong SAR, China

**Keywords:** public health: preventive medicine, health risk assessment, COVID-19, patient readmission, data science, artificial intelligence: machine learning and deep learning

## Abstract

**Background:**

Screening for frequent hospitalizations in the community can help prevent super-utilizers from growing in the inpatient population. However, the determinants of frequent hospitalizations have not been systematically examined, their operational definitions have been inconsistent, and screening among community members lacks tools. Nor do we know if what determined frequent hospitalizations before COVID-19 continued to be the determinant of frequent hospitalizations at the height of the pandemic. Hence, the current study aims to identify determinants of frequent hospitalization and their screening items developed from the Comprehensive Geriatric Assessment (CGA), as our 273-item CGA is too lengthy to administer in full in community or primary care settings. The stability of the identified determinants will be examined in terms of the prospective validity of pre-COVID-selected items administered at the height of the pandemic.

**Methods:**

Comprehensive Geriatric Assessments (CGAs) were administered between 2016 and 2018 in the homes of 1,611 older adults aged 65+ years. Learning models were deployed to select CGA items to maximize the classification of different operational definitions of frequent hospitalizations, ranging from the most inclusive definition, wherein two or more hospitalizations over 2 years, to the most exclusive, wherein two or more hospitalizations must appear during year two, reflecting different care needs. In addition, the CGA items selected by the best-performing learning model were then developed into a random-forest-based scoring system for assessing frequent hospitalization risk, the validity of which was tested during 2018 and again prospectively between 2022 and 2023 in a sample of 329 older adults recruited from a district adjacent to where the CGAs were initially performed.

**Results:**

Seventeen items were selected from the CGA by our best-performing algorithm (DeepBoost), achieving 0.90 AUC in classifying operational definitions of frequent hospitalizations differing in temporal distributions and care needs. The number of medications prescribed and the need for assistance with emptying the bowel, housekeeping, transportation, and laundry were selected using the DeepBoost algorithm under the supervision of all operational definitions of frequent hospitalizations. On the other hand, reliance on walking aids, ability to balance on one’s own, history of chronic obstructive pulmonary disease (COPD), and usage of social services were selected in the top 10 by all but the operational definitions that reflect the greatest care needs. The prospective validation of the original risk-scoring system using a sample recruited from a different district during the COVID-19 pandemic achieved an AUC of 0.82 in differentiating those rehospitalized twice or more over 2 years from those who were not.

**Conclusion:**

A small subset of CGA items representing one’s independence in aspects of (instrumental) activities of daily living, mobility, history of COPD, and social service utilization are sufficient for community members at risk of frequent hospitalization. The determinants of frequent hospitalization represented by the subset of CGA items remain relevant over the course of COVID-19 pandemic and across sociogeography.

## 1 Introduction

A small subset of the population disproportionately consumes medical resources, usually called “super-utilizers” (1). Frequent hospitalization among community members, especially community-dwelling elders, is a precursor to the proliferation of super-utilizers in the medical system and is a suggested target for community prevention programs (2). Naturally, identifying those with potential frequent hospitalizations is vital.

Numerous community studies have consistently shown that older adults in community who have unmet social needs are prone to frequent hospitalizations (see Tables 1, 2). Similarly, community studies have consistently shown that functional dependencies precipitate frequent hospitalizations among community members (see Tables 1, 2). Moreover, chronic illnesses, such as heart disease and COPD, along with comorbidities, medication use, geriatric depressive symptoms, cognitive status, and fall risk, have all been associated with frequent hospitalization (3, 4). However, these determinants, similar to unmet social needs or functional dependencies, have been examined in isolation in the literature and reported modest effect sizes. As a result, there is scant evidence regarding which determinants contribute more significantly to frequent hospitalizations, and knowledge about each determinant’s unique and combined contributions relative to all known determinants is limited.

**Table 1:** Characteristics of studies predicting frequent hospitalization outcome with selected instruments from CGA’s domains via non-EHR data source.

Study	Region/Country	Data Source	Definition of frequent hospitalization (≥A, T)	AUC	CGA domains			
					Medical	Social	Functional	Physical
Boult et al. (31)	US	In-person interview	4, 2	0.61	Diagnoses, falls history, UHS, URS	Socio-demographic	ADL, IADL, Cognitive Impairment	SRHS
Coleman et al. (32)	US	Home visit	4, 2	[0.69, 0.70]	Diagnoses, UHS	Socio-demographic	–	SRHS
Vojta et al. (33)	US	Mail survey	1, 0.5	[0.67, 0.68]	Diagnoses, UHS	Socio-demographic	–	SRHS
Jensen et al. (34)	US	Mail survey	1, 1	0.64	SRMS	Socio-demographic	ADL, IADL	Biometrics
Wagner et al. (35)	GM, UK, and SW	Home visit	1, 1	[0.62, 0.67]	Diagnoses, UHS	Socio-demographic	–	SRHS
Lyon et al. (36)	EN	Mail survey	1, 1	0.69	Diagnoses, Falls History, UHS, Medications	Socio-demographic	Cognitive Impairment	–
Mazzaglia et al. (37)	IT	In-person interview	1, 1.25	0.67	URS	Socio-demographic	ADL, IADL	Biometrics
Mosley et al. 2 (38)	US	Mail survey	1, 1	0.64	Diagnoses, UHS	Socio-demographic	–	SRHS
O’Caoimh et al. (39)	IR	In-person interview	1, 1	0.61	Diagnoses, SRMS	Socio-demographic	ADL	

A, # of admissions; T, time period (in years) during which frequent hospitalization is measured; US, United States; EN, England SW, Switzerland; GM, Germany; IR, Ireland; IT, Italy; CA, Canada; SG, Singapore; AUS, Australia; NL, Netherlands; CN, China; EHR, Electronic Health Records; SE, Sensitivity; SP, Specificity; ADL, Activity Daily Living; IADL, Instrumental Activity Daily Living; MMSE, Mini-Mental State Examination; UHS, Use of Hospital Services; URS, Use of Rehab Services; SRHS, Self-Rated Health Status; SRMS, Self-Rated Mental Status.

**Table 2:** Characteristics of studies predicting frequent hospitalization outcome with selected instruments from CGA’s domains via EHR data source.

Study	Region/Country	Data Source	Definition of frequent hospitalization (≥A, T)	AUC unless stated otherwise	CGA domains			
					Medical	Social	Functional	Physical
Cihi (40)	CA	EHR	1, 1.25	0.70	Diagnoses, UHS, URS	Socio-demographic	–	–
Tan et al. (41)	SG	EHR	4, 1	0.70	Diagnoses, UHS, LACE^70^	Socio-demographic		–
Pacala et al. (42)	US	EHR & Mail survey	1, 1	-	Diagnoses, UHS	Socio-demographic	–	SRHS
Kennedy et al. (43)	US	EHR and Mail Survey	1, 1	RR = 0.73–0.85	Diagnoses, UHS, Depression	Socio-demographic	Functional Impairment, ADL	SRHS, BMI
Longman et al. (44)	AUS	EHR, Mail Survey & Phone Survey	3, 1	OR = 0.36–4.76	Diagnoses, UHS	Socio-demographic	Fall History, ADL	SRHS
Meuleners et al. (45)	AUS	EHR	1, 1	HR = 1.07–5.78	Diagnoses, comorbidity, medications	Socio-demographic	Fall history	–
O’Leary et al. (46)	US	EHR and in-person interview	2 (30-day unplanned readmission), 1	–	Medical conditions/complications, UHS	Social and economic factors	Congenital disorder, Failure of self-management	Being previously healthy, Pain

A, # of admissions; T, time period (in years) during which frequent hospitalization is measured; US, United States; EN, England SW, Switzerland; GM, Germany; IR, Ireland; IT, Italy; CA, Canada; SG, Singapore; AUS, Australia; NL, Netherlands; CN, China; EHR, Electronic Health Records; SE, Sensitivity; SP, Specificity; ADL, Activity Daily Living; IADL, Instrumental Activity Daily Living MMSE, Mini-Mental State Examination; UHS, Use of Hospital Services; URS, Use of Rehab Services; SRHS, Self-Rated Health Status; SRMS, Self-Rated Mental Status; RR, relative ratio; OR, odds ratio; HR, hazard ratio.

Additionally, research examining the determinants of frequent hospitalizations during the COVID-19 pandemic is limited. Few studies have explored hospitalization patterns and readmission rates among inpatient populations during this critical period. Notably, these studies observed increased hospitalizations due to COVID-19 (5). COVID-19 patients with comorbid chronic conditions are at a higher risk of both initial hospitalization (6) and subsequent rehospitalization (7). In contrast, hospitalizations among non-COVID patients with chronic diseases, such as Chronic Obstructive Pulmonary Disease (COPD) or cardiovascular conditions, declined compared to the pre-pandemic levels (5). This reduction was attributed to improved self-management and adaptive health-seeking behaviors in response to pandemic-related public health and social measures (5). Consequently, it stands to reason that non-COVID patients with chronic illnesses hospitalized during the pandemic were more likely to lack effective self-management or have care needs that were too acute to engage in adaptive health-seeking behaviors that patients would otherwise exhibit during the pandemic, thereby increasing their risk of frequent hospitalization. Despite their potential contributions to preventive care, studies examining whether chronic illnesses and comorbidities have become stronger determinants of frequent hospitalizations during the pandemic, compared to the pre-COVID era, remain scarce. Furthermore, other determinants of frequent hospitalizations have not been systematically assessed to determine whether their effects differ between the pandemic and pre-pandemic periods.

However, identifying determinants of frequent hospitalizations through predictive models alone does not suffice to differentiate community-dwelling members at risk from those who are not. Instead, these determinants must first be operationalized as screening items and rigorously validated before inclusion as a single instrument. Currently, the literature lacks a validated instrument specifically designed for screening frequent hospitalization risk among community members. On the other hand, a tool has been shown to predict frequent hospitalization. The Comprehensive Geriatric Assessment (CGA) stands as the current gold standard for evaluating complex medical and social needs across multiple domains: medical, social, functional, and psychological etc. (8). Notably, the known determinants of frequent hospitalizations discussed earlier align with the CGA framework. Recent research has effectively employed the CGA to predict occurrences of frequent hospitalizations (9). Nevertheless, the resource-intensive nature of administering the CGA may not align with the available resources in community or primary care settings. In summary, while no validated tool has emerged from predictive model-identified determinants of frequent hospitalizations, the CGA, covering these determinants, holds promise as a screening tool for community members at risk. Practical considerations, however, warrant further exploration of alternative, resource-efficient screening practices.

Hence, we aim to deploy machine learning and deep learning methods to select individual items from CGAs administered in the home of community-dwelling older individuals. Specifically, CGA items were selected according to their unique and combined contributions to the older individuals’ frequent hospitalization profile. In addition, we explored operational definitions of frequent hospitalization that varied in temporal distributions of hospitalization events, reflecting the differences in care needs. By deploying and comparing learning models supervised by different operational definitions, we identified subsets of CGA items that maximized the accurate classification of frequent hospitalizations under each studied operational definition (hereafter, CGA short-forms). Furthermore, we developed and validated a random forest-based scoring system of frequent hospitalization risk from the CGA items selected by the best-performing learning model to enhance interpretability and applicability. Finally, to ensure the generalizability of our findings, we prospectively validated the scoring system in a separate cohort recruited during the peak of the COVID-19 pandemic from a district adjacent to where the full CGAs were administered.

## 2 Methods

### 2.1 Samples and data collection

Between 2016 and 2018, nurses and social workers administered CGAs in pairs at the homes of 1,611 older adults (65% female) aged 65+ (mean = 80.51, SD = 7.1). This cohort of 1,611 older adults was recruited from the clientele of one type of government-subsidized community service whose mandate is to provide those 65+ who can live independently in the community (as judged by licensed social workers) a network of support to provide care and concerns when needed. There were 61 community services of this type in total. In addition, to ensure the generalizability of our short-form CGA across time periods and contexts, we administered it to a prospective validation (10) cohort of 329 older adults [69% female; mean age 76.35 (SD = 8.02)], recruited between 2022 and 2023 from a series of community health assessment events held at the community centers in a district adjacent to the ones from which we sampled our 1,611 participants.

To ensure the privacy of participants during data collection, all individuals provided written informed consent after receiving a detailed explanation of the study’s purpose and procedures. Personal identifiers were replaced with unique alphanumeric codes to maintain anonymity throughout the research process. Data were collected using secure, encrypted devices, and all electronic files were stored on password-protected servers accessible only to authorized research personnel. Physical documents containing sensitive information were kept in locked cabinets within secured facilities. We strictly adhered to The Personal Data (Privacy) Ordinance of Hong Kong. The study protocol was reviewed and approved by the Research Ethics Board of Chinese University of Hong Kong to ensure compliance with ethical standards for human subject research. All data were used exclusively for this study’s objectives, and no identifiable information was disclosed in any reports or publications.

The CGA administered in the current study consisted of 19 standardized instruments, encompassing 206 individual items and generating 19 total scores. Individual items from all standardized instruments in the CGA were coded as ordinal variables, while their respective total scores were coded as interval scales. Additionally, the open-ended interview questions were coded into 127 binary variables (yes/no responses) or, when dealing with categorical variables with multiple possible values, into sets of dummy variables. The binary and dummy variables coded from the open-ended questions represented participants’ unmet needs across different domains, including medical history, service utilization history (medical and social), and medication history. For details on the methods we have adopted to code and pre-process individual items and total scores from different instruments to build a feature pool, and to train and validate machine learning models built from such a feature pool, please refer to Choi et al. (11).

### 2.2 The development and validation of short-form CGA in a cohort of 1,611 clients of an older adult service

#### 2.2.1 Operational definition of frequent hospitalizations

In the current study, frequent hospitalization outcomes were derived from participants’ self-report of the dates of their past hospitalizations over the 2 years prior to the CGAs.

Specifically, as previous studies have shown (12, 13), the closer the hospitalizations were to when the assessment of care needs was performed, the greater the care needs the individuals had, even compared to those hospitalized with the same frequency, but spread over the entire study period. Hence, not only did the current study operationally define frequent hospitalization as two or more hospitalizations over a two-year period in accordance with the literature on frequent hospitalization (see Tables 1, 2), but it also examined different operational definitions with respect to whether the self-report hospitalizations occurred in the year immediately before when the participants were asked to recall the hospitalization events (namely “the 2nd year”) or did the hospitalization occur during the year prior to the one immediately before when the assessment was conducted (“the first year”).

Here, depending on whether hospitalizations occurred during the first or second year (or both), four operational definitions of frequent hospitalizations were derived from a progressively less inclusive temporal distribution of their two or more hospitalization events. In particular, our first and most inclusive operational definition of frequent hospitalization requires only two or more hospitalizations to occur at any time over the two-year prior to the assessment. On the other hand, the second and incrementally more restrictive operational definition required that at least one hospitalization occurred in year one. The third operational definition was even more restrictive, which included the scenarios of (1) having two or more hospitalizations in year one, regardless of year two’s hospitalization pattern, or (2) having exactly one hospitalization in each of the 2 years. Finally, the most restrictive operational definition of frequent hospitalization studied here required that two or more hospitalizations occur at year two, regardless of the hospitalization pattern during the 1st year.

#### 2.2.2 Dimension reduction of CGA with machine learning and deep learning algorithms

The four increasingly stringent operational definitions of frequent hospitalizations were used as supervisory outcomes in the training and 10-fold cross-validation of four machine learning algorithms: LASSO, Decision Tree, AdaBoost, and DeepBoost. The four algorithms were selected to represent a wide spectrum of complexity, parametricity, and their ability to handle multicollinearity—which is particularly important in the current study. Not only are individual standardized instruments differentially related to one another, but the items within each instrument and their respective total scores are also highly correlated. At one end of the spectrum lies LASSO, a simple, parametric linear model that has limited capacity to model complex non-linear relationships or handle multicollinearity effectively. At the other end is DeepBoost, which integrates deep learning architectures into the boosting framework, enabling it to capture highly complex and non-linear relationships, hierarchical pattern and multicollinearity in the data. In the middle of the spectrum are Decision Tree and AdaBoost. Decision Tree is a non-parametric model that outperforms linear models like LASSO in representing non-linear relationships, though with moderate complexity. AdaBoost, on the other hand, captures moderate to high levels of complexity by combining multiple weak learners (typically shallow decision trees) into an ensemble. In addition, all four algorithms include feature selection mechanisms: LASSO utilizes coefficient shrinkage, Decision Tree reduces impurity, AdaBoost emphasizes error correction across weak learners, and DeepBoost derives feature importance from network weights. DeepBoost’s ability to capture feature importance is particularly robust when modeling complex interactions among features.

The performance of the studied algorithms were examined via 10-fold validation, and the validated performance is parameterized and compared in terms of the area under the receiver operating characteristic curve (AUC). The 95% confidence interval (95% CI) and *p*-value of the AUCs resulting from 10-fold validation of the algorithms were calculated based on the formular documented in Zhou et al. (14). In addition, the interpretation of the AUCs reported in the current study was also based on Zhou et al. (14), where by the model’s discriminatory performance was considered poor if the AUC was <0.70, acceptable when AUC = 0.70 to <0.80, excellent when AUC = 0.80–0.90, and outstanding when AUC >0.90.

Furthermore, Spearman’s rank correlations were performed to compare the items selected, and the order in which items were selected, by the best-performing algorithm’s four operational definitions of frequent hospitalization outcomes were examined.

#### 2.2.3 Risk scoring the short-form CGA with a machine learning-based algorithm

Selecting a small set of CGA items with the highest feature importance is not sufficient to fulfil the CGA’s “intended clinical use” (15). For these selected CGA items to effectively screen community-dwelling older adults for frequent hospitalization risk as intended, they must also be assigned appropriate weights and scored. Traditionally, scoring for screening tools has been based on regression models. However, regression-based approaches are not well-suited to handle the complex, non-linear relationships captured by machine learning-driven feature selection processes. In fact, applying regression-based scoring could oversimplify—or “flatten”—the nuanced interactions among features, thus undermining the very reason why machine learning models were deployed in feature selection.

To address this challenge, the current study employs a state-of-the-art machine learning-driven scoring algorithm that specializes in translating complex, non-linear relationships between features and outcomes into an interpretable and implementable scoring system (16, 17). Specifically, outcome-differentiating weights were assigned to each response level of the selected CGA items to ensure that the scoring system remains both clinically meaningful and robust enough to handle the intricacies inherent in the data, and thereby enhancing its utility as a screening tool for identifying older adults at risk of frequent hospitalization.

Notably, only the most inclusive operational definition of frequent hospitalization—defined as two hospitalization events over the course of 2 years—was used in the construction of the scoring system from CGA items that the best-performing learning models had selected. This decision was based on the expectation that the risk of frequent hospitalization among community-dwelling older adults in the general population is relatively low. By focusing on the broadest definition, we aimed to capture a meaningful signal while accounting for the lower baseline hospitalization rates typically observed in the community population.

### 2.3 Prospective validation of the scoring system developed from the short-form CGA with a cohort of 329 older adults during the COVID-19 pandemic

To ensure the generalizability of our short-form CGA and the machine learning-driven scoring system derived from it, we performed prospective validation under conditions different from those under which the CGA was initially abbreviated and its scoring system was devised. Prospective validation provides a more rigorous assessment of model robustness by testing performance under conditions that differ from those during development, capturing the potential impact of temporal and contextual shifts that traditional validation methods may fail to account for. In the current study, for the purpose of prospective validation, a sample was recruited from a district adjacent to the model-building cohort. Different from the original cohort of 1,611 older adults recruited from the clientele of a type of government-subsidized community service, the prospective validation cohort was recruited through venue-based sampling of older adults who attended health assessment events hosted by the participating NGO. In addition, the validation cohort’s clinical and functional status, as well as frequent hospitalization outcome, were assessed by community workers and healthcare professional trainees (who are commonly responsible for in-take assessments in local NGO context). In contrast, the assessment of the 1,611 older adult clients’ clinical and functional statuses and frequent hospitalization outcomes were performed by licensed nurses and social workers who initially performed CGAs in the home of the 1,611 clients of the government-subsidized community service. Finally, the validation cohort was recruited during the COVID-19 pandemic between 2022 and 2023, rather than during the same period when the initial cohort of 1,611 was recruited between 2016 and 2018 to develop the short-form of CGA. COVID-19 has had disruptive effects on healthcare systems worldwide, impacting their organization, utilization, and practice culture, while simultaneously altering the risk, composition, severity, and chronicity of diseases within communities. Therefore, by testing the scoring system derived from a model built and validated before the pandemic using data collected during its peak, we subjected our scoring system to the most stringent examination. This approach ensures that the ensemble of identified factors—and their relative contributions to frequent hospitalization outcomes—can withstand fundamental changes like those introduced by COVID-19 and remain as valid during the peri-COVID period as it was before the pandemic.

Members of the prospective validation cohort were considered at risk of frequent hospitalization if they scored above the cut-off value of the scoring system derived from items of the short-form CGA. The prospective validation cohort’s self-reported frequency of hospitalizations during the 2 years prior to the date of assessment was ascertained as the outcome for validating the risk status assigned by our risk-scoring system. A logistic regression model was used to validate the status assigned by our risk-scoring system against the self-reported frequent hospitalization outcome. The performance of the logistic model was measured in terms of its AUC. The AUC’s 95% confident interval and *p*-value were also reported.

## 3 Results

The following are the (1) performance of different learning algorithms deployed to select items from CGA under the supervision of different operational definitions of frequent hospitalizations; (2) rank-ordering of the CGA items selected by different operational definitions of frequent hospitalizations; (3) Scoring of CGA items selected by the best-performing learning algorithm to maximize the differentiability of those who were hospitalized twice or more over 2 years from those who were not; and (4) prospective validation of the scoring system with a cohort of 329 sampled from a different population during the pandemic between 2022 and 2023.

### 3.1 The prevalence of each operational definition of frequent hospitalization among the 1,611 community service clients

Of the 1,611 clients, 225 (14.0%) were hospitalized twice or more during the 2 years prior to the assessment (i.e., the first operational definition). Of the 225 clients who met the first operational definition, 200 were hospitalized at least once in the first year (the second operational definition), of which 193 also met the third operational definition. Finally, 158 of those who met the third operational definition also met the fourth operational definition, whereby two or more hospitalizations occurred in year two, regardless of the hospitalization pattern during the 1st year.

### 3.2 The performance of learning model-driven selection of CGA items

**Figure 1:**
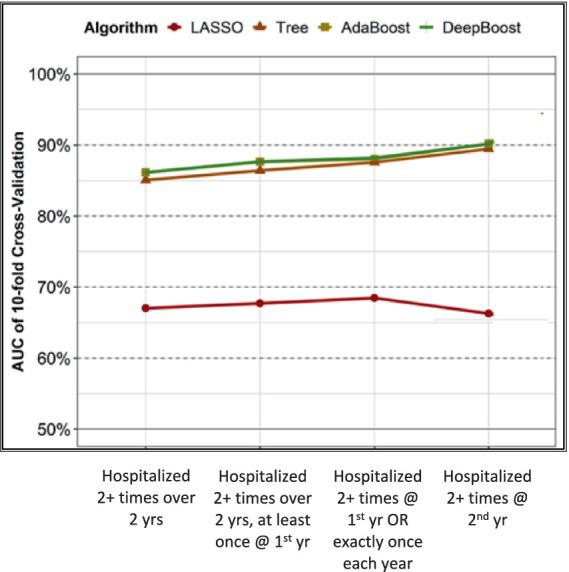
Performance of LASSO, tree, AdaBoost, and DeepBoost on classifying 4 operational definitions of frequent hospitalization.

Figure 1 shows the performance of the four learning algorithms under the supervision of each of the four operational definitions of frequent hospitalizations as outcomes. Our 10-fold validation analyses revealed that DeepBoost and AdaBoost consistently outperformed decision trees and LASSO across all operational definitions. Specifically, for the four increasingly exclusive operational definitions, DeepBoost and AdaBoost achieved AUCs of 0.87 (95% CI: 0.84–0.90; *p* < 0.001), 0.88 (95% CI: 0.85–0.91; *p* < 0.001), 0.88 (95% CI: 0.85–0.91; *p* < 0.001), and 0.90 (95% CI: 0.87–0.93; *p* < 0.001), respectively. On the other hand, the decision tree algorithm yielded lower AUCs of 0.85 (95% CI: 0.82–0.88; *p* < 0.001), 0.86 (95% CI: 0.83–0.89; *p* < 0.001), 0.87 (95% CI: 0.84–0.90; *p* < 0.001), and 0.89 (95% CI: 0.86–0.92; *p* < 0.001), respectively. The LASSO algorithm achieved the lowest AUCs of 0.67 (95% CI: 0.63–0.71; *p* < 0.001), 0.68 (95% CI: 0.64–0.72; *p* < 0.001), 0.68 (95% CI: 0.64–0.73; *p* < 0.001), and 0.66 (95% CI: 0.61–0.71; *p* < 0.001), respectively.

AdaBoost and DeepBoost were equally superior to LASSO and decision tree models in modeling frequent hospitalization outcomes. Therefore, we focused on comparing and scoring only the CGA items selected by DeepBoost in the subsequent analysis because its feature selection mechanism best captures the complex interactions among features and between features and outcomes.

### 3.3 Rank-ordering of CGA items selected by each operational definition

Table 3 shows side by side for each studied operational definition-supervised ranking-ordering of features (i.e., CGA items) were selected from the full CGA. Each column labeled by its corresponding operational definition reports the rank ordering of features selected under its supervision. Our analyses revealed that the items selected by all four operational definitions were similar, despite the differences in the order with which they were selected. For example, the number of medications prescribed and the need for assistance with emptying the bowel (an item on a CGA’s instrument, Activity Daily Living) were consistently the features assigned the highest and second-highest rankings, respectively, across all four operational definitions. Nevertheless, even when all four operational definitions ranked the same three items from Instrumental Activity Daily Living (requiring assistance with housekeeping, transportation, and laundry) as their respective top 10, the items were selected as model features in a different order. However, items selected in the top 10 by one operational definition could be left unselected by another. For example, while an older adult’s reliance on walking aids, usage of additional social services, and being prescribed 3+ medications were ranked in the top 10 by the first three operational definitions, they were not selected by the fourth operational definition, which is the most restrictive in terms of requiring 2+ hospitalizations during the year immediately prior to the assessment (i.e., year two). In addition, when the rank orders of features selected under the supervision of the first three operational definitions were significantly correlated with one another (Spearman’s rhos = 0.82–0.95), the rank order of features selected under the fourth operational definition was not significantly correlated with any other operational definitions’ rank orders.

**Table 3:** DeepBoost-selected CGA features by rank of feature importance in four operational definitions of frequent hospitalization.

Features\Rank by feature importance	Operational definition of frequent hospitalization			
	Definition1: Two or more hospitalizations during the two-year period	Definition2: Two or more hospitalizations during the two-year period, and at least once in year one	Definition3: Two or more hospitalizations during year one, regardless of year two’s hospitalization pattern; OR hospitalized exactly once each of the 2 years	Definition4: Two or more hospitalizations during year two, despite year one’s hospitalization pattern
Number of medications	1	1	1	1
Required assistance with emptying bowel (ADL)	2	2	2	2
Required assistance with housekeeping (IADL)	6	5	3	3
Required assistance with transportation (IADL)	4	6	4	4
Required assistance with laundry (IADL)	8	7	5	6
Ever diagnosed with COPD	9	11	11	12
Reliance on walking aids	5	4	7	–
Additional social service usage	7	3	6	–
Presence of polypharmacy (3+)	3	9	8	–
Ability to balance while standing (Tinetti assessment)	10	8	9	–
Required assistance with medications (IADL)	–	10	12	7
Required assistance with shopping (IADL)	–	12	10	10
Dropped many activities and interests (GDS)	–	–	–	5
Bladder assistance (ADL)	12	–	–	11
Required assistance with food preparation (IADL)	–	–	14	9
Ever diagnosed with stroke	–	–	15	8
Required assistance with finance (IADL)	–	13	13	–
Ever diagnosed with heart disease	11	–	–	–

ADL, Barthel Index of Activities of Daily Living; IADL, Instrumental Activities of Daily Living; GDS, Geriatric Depression Scale; COPD, Chronic obstructive pulmonary disease.

### 3.4 Scoring of selected CGA items to maximize the differentiability of those who were hospitalized twice or more over 2 years from those who were not

Using the data collected from our cohort of 1,611 clients of an older adult service, Table 4 shows the descriptive statistics of the CGA items that our machine learning model selected and assigned scores relative to each selected feature’s response level. As Table 4 shows, the older persons with more medications, higher dependency, and a greater prevalence of physical and mental illnesses received greater scores toward the cutoff value of 49, beyond which the clients were deemed at risk of frequent hospitalization. In particular, dependency on emptying the bowel, shopping for daily necessities, inability to balance while standing on one’s own, and having 10 or more medications were assigned the largest scores toward the overall sum compared to other response levels. Non-reference response levels for all selected items were assigned scores that corresponded to their unique contributions to the outcome, except for the feature of having 3+ medications (polypharmacy), which failed to make any additional contribution to the outcome beyond what the number of medications contributed when the count measure was categorized into specific ranges that optimized the feature outcome prediction.

**Table 4:** Descriptive statistics of the Comprehensive Geriatric Assessment (CGA) items selected by our machine learning model and the scores our model assigned to each feature’s response level.

Selected CGA items (as model features)	Response levels	Distribution of the cohort of 1,611 older adult across each selected feature’s response level	Scores assigned to each selected feature’s response level
1. Number of medications	<1	11%	0
	[1,6)	61%	6
	[6,10)	23%	7
	≥10	5%	9
2. Required assistance with emptying bowel (ADL)	Dependent	1%	19
	Partly dependent	3%	3
	Independent	97%	3
3. Required assistance with housekeeping (IADL)	Dependent	46%	5
	Partly dependent	30%	5
	Independent	24%	0
4. Required assistance with transportation (IADL)	Dependent	6%	2
	Partly dependent	11%	2
	Independent	83%	1
5. Required assistance with laundry (IADL)	Dependent	6%	5
	Partly dependent	8%	1
	Independent	87%	1
6. Ever diagnosed with COPD	No	94%	0
	Yes	6%	8
7. Reliance on walking aids	No	60%	0
	Yes	40%	1
8. Additional social service usage	No	71%	0
	Yes	29%	2
9. Ability to balance while standing (Tinetti assessment)	Dependent	1%	9
	Partly dependent	14%	9
	Independent	86%	0
10. Required assistance with managing medications (IADL)	Dependent	2%	0
	Partly dependent	9%	3
	Independent	89%	3
11. Required assistance with shopping (IADL)	Dependent	4%	11
	Partly dependent	7%	0
	Independent	89%	0
12. Dropped many activities and interests (GDS)	No	87%	0
	Yes	13%	5
13. Bladder assistance (ADL)	Dependent	1%	7
	Partly dependent	8%	4
	Independent	91%	0
14. Required assistance with food preparation (IADL)	Dependent	7%	3
	Partly dependent	5%	3
	Independent	88%	0
15. Ever diagnosed with stroke	No	91%	0
	Yes	9%	3
16. Required assistance with managing finance (IADL)	Dependent	4%	2
	Partly dependent	6%	2
	Independent	90%	0
17. Ever diagnosed with Heart Disease	No	80%	0
	Yes	20%	4

### 3.5 Prospective validation of the scoring system

Of the 329 older adults recruited from the community during health assessment events hosted by the studied NGO, 13.1% were hospitalized at least twice over the course of 2 years. Table 5 shows the descriptive statistics of the risk-scoring system applied to a community sample of 329 older adults. While the percentages of the sample’s self-reports of frequent hospitalization were similar, it is shown in Table 5 that despite the sample of 329 older adults recruited from health assessment events, is significantly older than the sample of 1,161, they required less assistance in instrumental activities of daily living, such as housekeeping, laundry, and food preparation, and were less likely to report a history of heart diseases and have fewer medications compared to the 1,611 clients of community service. Nevertheless, when the risk-scoring system developed from the 1,611 cohort was prospectively validated with data on predictors and outcomes ascertained from the prospective validation cohort of 329, the logistic model was performed at the level of AUC = 0.82 (95% CI = 0.74–0.90; *p* < 0.001).

**Table 5:** Descriptive statistics of Comprehensive Geriatric Assessment (CGA) items selected by our pre-COVID-19 machine learning model, administered to a community sample of 329 older adults during the COVID-19 pandemic.

Selected CGA items (as model features)	Response levels	Distribution of the cohort of 329 older adult across each selected feature’s response level
1. Number of medications	<1	19%
	[1,6)	75%
	[6,10)	4%
	> = 10	3%
2. Required assistance with emptying bowel (ADL)	Dependent	2%
	Partly dependent	5%
	Independent	92%
3. Required assistance with housekeeping (IADL)	Dependent	5%
	Partly dependent	22%
	Independent	73%
4. Required assistance with transportation (IADL)	Dependent	3%
	Partly dependent	8%
	Independent	89%
5. Required assistance with laundry (IADL)	Dependent	3%
	Partly dependent	6%
	Independent	91%
6. Ever diagnosed with COPD	No	90%
	Yes	10%
7. Reliance on walking aids	No	68%
	Yes	32%
8. Additional social service usage	No	24%
	Yes	76%
9. Ability to balance while standing (Tinetti assessment)	Dependent	2%
	Partly dependent	14%
	Independent	84%
10. Required assistance with managing medications (IADL)	Dependent	2%
	Partly dependent	7%
	Independent	91%
11. Required assistance with shopping (IADL)	Dependent	2%
	Partly dependent	5%
	Independent	92%
12. Dropped many activities and interests (GDS)	No	65%
	Yes	35%
13. Bladder assistance (ADL)	Dependent	2%
	Partly dependent	11%
	Independent	88%
14. Required assistance with food preparation (IADL)	Dependent	3%
	Partly dependent	5%
	Independent	92%
15. Ever diagnosed with stroke	No	89%
	Yes	11%
16. Required assistance with managing finance (IADL)	Dependent	2%
	Partly dependent	5%
	Independent	92%
17. Ever diagnosed with Heart Disease	No	84%
	Yes	16%

## 4 Discussion

With a 10-fold validation AUC of 0.87–0.90, our DeepBoost algorithm classified those who met different criteria of frequent hospitalization prior to COVID-19 with only 17–23 CGA items selected based on feature importance computing from the network weights. The selected items included the presence of selected chronic illnesses and polypharmacy, as well as participants’ functional and psychosocial statuses. In addition, when a scoring system was derived from the selected CGA items and applied to a prospective validation sample recruited during the height of COVID-19, it remained valid with an AUC of 0.82.

The findings of the current study add value to the literature by identifying the determinants of frequent hospitalization, as reviewed in Tables 1, 2. While it has been shown that the more recent the previous hospitalization, the greater the risk of re-hospitalization, the temporal dimension has never been factored into previous studies in modeling frequent hospitalizations and identifying their determinants. Instead, previous studies either examined hospitalization counts within a single year or their average counts when hospitalizations spanning multiple years were considered. In contrast, this study operationally defined frequent hospitalizations with respect to the timing between when the assessments were performed and when hospitalizations occurred.

To our knowledge, as summarized in Tables 1, 2, the majority of studies investigating the determinants of frequent hospitalization have been conducted in the United States and the United Kingdom, with Asian populations rarely studied. This is significant because populations in Asia are aging more rapidly, and conclusions drawn from studies in Western countries may not be applicable to Asian societies. Therefore, the current study adds value to the literature by identifying the determinants responsible for frequent hospitalizations of older adults in rapidly aging Hong Kong, both before and during COVID-19.

In addition, our analyses revealed that non-parametric machine learning models (especially DeepBoost) outperformed parametric models (such as LASSO) across all operational definitions studied. Our results are consistent with previously published studies showing that linear model-based psychometric analysis has limited validity when the data are of high dimension, imbalanced, and complex. Consequently, the nonparametric learning model is best suited for reducing the dimension of an assessment package as intrinsically complex as CGA by selecting its intricately interrelated items according to their marginal (singly or combined) contributions to the studied outcomes. To our knowledge, only one recent study (11) compared the validity of linear model-based psychometric analysis with supervised machine learning in the dimension reduction of data collected from multiple interrelated standardized instruments for assessing low-prevalence health outcomes (clinical depression diagnosis among university students). Specifically, Choi et al. (11) first extracted latent representational layers from what was shared among different instruments administered, and subsequently estimated the importance of individual items according to their respective strengths with these shared latent layers. Choi et al. (11) also concluded that non-parametric learning models outperformed traditional linear model-based psychometric analyses in terms of dimension reduction.

Hence, findings of the current study has also contributed to the effort in abbreviating the CGA by addressing the research gaps in linear model-based psychometrics and lacking clinically relevant outcome that led a recent literature review on the matter to conclude that there is little evidence to promote the use of one (CGA items) over another or a combination of their components” (18). While being a gold standard in multidimensional needs assessment, CGA is too resource-intensive to administer (19, 20). Hence, instead of administering the CGA in its entirety, a “two-step approach” (19) is advised, wherein an abbreviated version of the CGA can first be administered to screen for high-risk individuals, followed by administering the full-length CGA to those who were screened positive of risk. Consequently, several short-forms of CGA have been published (21–27). However, a recent review noted that these short forms’ “psychometric properties,” such as the reliability and internal consistency established using classical statistics such as linear model-based principal component analysis, “as well as their impact on clinically relevant outcomes, have not been thoroughly examined. Consequently, there is little evidence to promote the use of one over another or a combination of their components” (18).

One of the two research gaps found among the studies that abbreviated CGA was that the psychometric properties of short forms yielded from abbreviating the CGA remained unexamined. Furthermore, the omission may also result from the incompatibility between the established linear-model-based psychometric methodology and the nature of the data that CGA yields. Specifically, the traditional psychometric approach to dimension reduction (28) cannot handle high-dimensional, imbalanced, or complex data (29, 30). However, data resulting from CGAs administered in community settings are (1) high-dimensional (as CGA assesses care needs across the medical, social, functional, and physical domains thoroughly), (2) imbalanced (as extreme values among CGA items are rare given the low prevalence of high-risk individuals in primary care settings), and (3) complex (as the strength of relationships is different between different pairs of CGA domains, among different instruments that fall within the same domains, and among different items that belong to the same instruments).

The second research gap identified was that studies advancing short-forms of CGA did not thoroughly examine their impact on any clinically relevant outcomes (18) [even though criterion validation with non-clinical outcomes had been performed (22, 25, 26)], which is in stark contrast with the clinical outcome-driven development of CGA. Specifically, CGA was originally developed to assess the risk of frequent hospitalizations among medically frail older adults (20, 22). Hence, frequent hospitalizations, often defined in the literature as two or more hospitalizations in a year on average, is the most studied outcome of CGA (8). However, frequent hospitalization was never examined as the predicted outcome of any published short forms of CGA, nor was frequent hospitalization the criterion to which the published short forms of CGA were validated against. As a result, the current study bridged the research gaps of lacking formal psychometrics and clinically relevant outcomes in developing short forms for CGA with a supervised machine learning approach to dimension reduction. Consequently, the current study achieved the objective of providing evidence for “the use of one over and other or a combination of CGA’s components” (9).

In addition to abbreviating the CGA, the current study applied a machine learning-driven scoring method to assign relative weights to each selected CGA item. This approach captures the complex and non-linear relationships among the items included in the resulting screening tool. Assigning weights and developing a risk-scoring system enhances the interpretability of the relative importance of selected items, thereby facilitating clinical and public health practices. The interpretability of the scores assigned to each item with respect to frequent hospitalization risk allows us to examine the generalizability of the items selected by the deep learning algorithm, which is known for its tendency to overfit. Moreover, being able to generalize from the period prior to the pandemic to its peak is particularly critical for testing the validity of our model. COVID-19 has not only transformed our medical system but has also left many community members with long-term effects that further exacerbate their pre-existing chronic conditions and poor functionality. Therefore, demonstrating that our risk-scoring system remains valid across such fundamentally different contexts underscores its robustness and practical utility.

This study had several limitations. First, our sample of 1,161 older adults consisted of clients from a standard government-subsidized community service catering to individuals aged 65 and above who lived independently with low clinical and social needs; eligibility was determined by licensed health professionals. Consequently, the sample is not representative of the heterogeneous community-dwelling older adult population. Future research on population-based frequent hospitalization risk screening could benefit from developing tools using population-representative samples. Nevertheless, our sample is comparable to clients encountered in primary and community care settings for which the abbreviated Comprehensive Geriatric Assessment (CGA) was intended. Therefore, the abbreviated CGA developed here adheres to the advice that risk assessment and modeling tools should be built according to their “intended clinical use” (15).

Second, the prospective validation cohort—sampled during health assessment events amid COVID-19—was also not representative of the heterogeneous community-dwelling older adult population. Community members who recognized the need for health assessments and were motivated to attend such events were not representative of a population of community dwelling older adults. Additionally, findings from a sample recruited during the pandemic may not generalize to non-pandemic times. However, given the differences between the model development and validation cohorts in sample characteristics, pandemic presence, and geographical location, it can be inferred that the resulting risk-scoring tool is robust against changes in sample, time, and location.

Finally, the number of hospitalization events used as the supervisory outcome was based on participants’ retrospective recall of hospitalizations within the 2 years prior to CGA administration. Although these self-reported events were verified by home-visiting healthcare professionals through reviews of hospital discharge summaries, lapses in memory or misplaced records may have introduced errors into the analysis. To improve the validity of frequent hospitalization measurements, future studies should consider direct access to electronic health records (EHRs).

## 5 Conclusion

In conclusion, the risk-scoring system we developed from the short-form CGA can be applied to screen various populations of older adults for frequent hospitalization risk. It also identifies a minimal set of underlying care needs parsimoniously (16), enabling targeted tertiary prevention of frequent hospitalization. For example, community services can address the dependency needs of older adult residents on specific instrumental activities of daily living to reduce their likelihood of frequent hospitalization. Future research should aim to increase the diversity and size of validation samples to examine the scoring system’s validity under a broader range of conditions and to identify commonalities and differences among the diverse older adult populations within our demographics.

## Data Availability

The datasets generated and analyzed during the current study are not publicly available as they are the property of the partnering NGO, which is bounded by Hong Kong’s Personal Data (Privacy) Ordinance (Cap. 486) (PDPO), including, but not exclusive to, PDPO’s Guidance Note in Cross-border Data Transfer. In addition, the Research Ethics Committees of the PI’s institution do not allow a third-party transfer of social service client information. Nor do the Ethics Committees permit study PI to make public the social service clients’ information.
